# Population Aging and Migrant Workers: Bottlenecks in Tuberculosis Control in Rural China

**DOI:** 10.1371/journal.pone.0088290

**Published:** 2014-02-03

**Authors:** Sumedh Bele, Wei Jiang, Hui Lu, Hua You, Hong Fan, Lifang Huang, Qungang Wang, Hongbing Shen, Jianming Wang

**Affiliations:** 1 Department of Social Medicine and Health Education, School of Public Health, Nanjing Medical University, Nanjing, P. R. China; 2 Department of Epidemiology and Biostatistics, School of Public Health, Nanjing Medical University, Nanjing, P. R. China; 3 Department of Tuberculosis, Center for Disease Control and Prevention of Zhangjiagang, Suzhou, P. R. China; Universitá Cattolica del S. Cuore, Italy

## Abstract

**Background:**

Tuberculosis is a serious global health problem. Its paradigms are shifting through time, especially in rapidly developing countries such as China. Health providers in China are at the forefront of the battle against tuberculosis; however, there are few empirical studies on health providers' perspectives on the challenges they face in tuberculosis control at the county level in China. This study was conducted among health providers to explore their experiences with tuberculosis control in order to identify bottlenecks and emerging challenges in controlling tuberculosis in rural China.

**Methods:**

A qualitative approach was used. Semi-structured, in-depth interviews were conducted with 17 health providers working in various positions within the health system of one rural county (ZJG) of China. Data were analyzed based on thematic content analysis using MAXQDA 10 qualitative data analysis software.

**Results:**

Health providers reported several problems in tuberculosis control in ZJG county. Migrant workers and the elderly were repeatedly documented as the main obstacles in effective tuberculosis control in the county. At a personal level, doctors showed their frustration with the lack of new drugs for treating tuberculosis patients, and their opinions varied regarding incentives for referring patients.

**Conclusion:**

The results suggest that several problems still remain for controlling tuberculosis in rural China. Tuberculosis control efforts need to make reaching the most vulnerable populations a priority and encourage local health providers to adopt innovative practices in the local context based on national guidelines to achieve the best results. Considerable changes in China's National Tuberculosis Control Program are needed to tackle these emerging challenges faced by health workers at the county level.

## Introduction

The countdown to the 2015 deadline for the Millennium Development Goals (MDG) has already begun [1]. Although the MDG target to halt and reverse the tuberculosis (TB) epidemic by 2015 has already been achieved [2], the global rate of decline in the incidence of TB is less than 1% per year which makes it impossible to achieve TB elimination by 2050 until the gaps in worldwide TB control are identified and resolved [3], [4]. Currently, one third of the global population is infected with the pathogen, *Mycobacterium tuberculosis*, and there were 1.3 million deaths due to TB in 2012 [2]. Thus, 20 years after the WHO declared TB a global emergency [5], TB remains a major threat to global health today.

In 2000, China had 5 million active TB cases, resulting in 130,000 deaths annually [6]. During the last decades, China has made remarkable progress in controlling TB by cutting its prevalence in half, reducing its incidence by 3.4% per year and reducing the TB-induced mortality rate by 80%; however, with approximately 0.86 million newly notified tuberculosis cases in 2012 [2], China still has the second highest burden of TB after India.

Hospitals play a crucial role in TB control. Public hospitals are the largest healthcare providers in China, but usually they are not part of the National Tuberculosis Control Program (NTP). To achieve TB control targets, China has applied a public-public mix approach (PPM) between public hospitals and TB control programs. In China, there are three models of collaboration (i.e., the dispensary, specialist and integrated models) between hospitals and the Center for Disease Control and Prevention (CDC), depending on their roles and responsibilities in TB control. Under the integrated model, TB care is integrated into tertiary-level hospital care by setting up a clinic to provide services for TB patients. Such hospitals are recognized as ‘designated hospitals.’ All primary and secondary health facilities in the area have to refer suspected or confirmed TB cases to this clinic for confirmation of diagnosis, treatment and follow-up. The CDC is responsible for the public health aspect of TB care, which includes health promotion, case reporting, supervision, and training for health providers. A nationwide, internet-based disease reporting system, the China Information System for Disease Control and Prevention (CISDCP), helps CDC staff obtain updated information about suspected and diagnosed TB cases from all the hospitals in the county. If the referred patients fail to attend designated hospitals within 3 days, CDC staff have to track those patients using the information uploaded to CISDCP by the referring hospitals.

Rapid urbanization and development of rural areas have changed the priorities of health systems as new challenges are transforming paradigms of TB control in China. The traditional vertical approach of TB control programs has been criticized for ignoring local context because it focuses on short-term and target-oriented activities rather than capacity building. Chinese NTP focuses more on TB cases to control further transmission than individuals' rights and the needs of such patients [3], [7]. Based on research and experience from the implementation of such programs, there has been a shift in perspectives from a top-down control approach to bottom-up strategies, emphasizing patient-centered care, community involvement and coordination between ‘actors’ at different levels for TB control [8].

Health care providers at the county level are at the forefront of TB control efforts; however, there are few studies in China reporting their perspectives to identify challenges in TB control at the county level. This paper reports emerging challenges for TB control as perceived by health care providers in one rural county in China.

## Methods

### Ethical considerations

The Institutional Review Board (IRB) of Nanjing Medical University approved the study. Written informed consent was obtained from all participants.

### Design and data collection

This study employed a qualitative approach to explore health providers' perceptions of TB control in one rural county (ZJG) in China from March to May, 2013. ZJG is a county-level city situated in the middle-eastern part of China and has an estimated population of 1.3 million people. County-level cities differ from regular ‘cities’ because they usually contain some rural areas. The CDC, designated hospitals and community health centers are located in urban areas while township hospitals and village health stations are located in rural areas of the county. Since 2004, ZJG county has adopted an integrated model of TB control. In this model, clinical care for TB is provided by the People's General Hospital of ZJG county serving as a designated hospital under the leadership of ZJG county's CDC. TB control programs involve health providers at all levels of the health system. In ZJG county, 14 health providers are involved in TB control activities at the designated hospital, and 4 health managers work in the department of TB control at the CDC. In addition, there are one or two doctors at each secondary level hospital responsible for diagnosis, referral and monitoring of TB patients.

A qualitative approach was used in this study. Data were gathered following face-to-face in-depth interviews with 17 key respondents (Table 1) who were intentionally selected as representatives of other health providers working at the same position in other health facilities in ZJG county. A semi-structured topic guide was developed, initially using the ‘grounded theory’; it was revised following two pilot interviews and was adapted continuously after each interview. Participants were asked about the current TB situation in their area, their opinions about the current TB control program, and the types of challenges they were facing in controlling TB. All the interviews were conducted in Mandarin by a researcher trained in qualitative data collection methods. Participants' responses guided the interviewer in deciding when and how to probe the emergent themes. All the interviews were arranged by the ZJG CDC and conducted at the workplace of the interviewees. All participants were informed about the general subject of the study during an informed consent procedure prior to the interviews. All the interviews were audio recorded with participants' permission, transcribed verbatim and translated into English. Some of the interviewees were contacted again to clarify unclear points. Informants' anonymity was guaranteed.

**Table 1:** Sampling for in-depth interviews in ZJG county.

Organization	Position	Number
Center for Disease Control and Prevention (CDC)	Director	1
	Head of TB section	1
	Staff of TB section	1
	Radiologist at CDC health clinic	1
	CDC staff at designated hospital	1
Designated hospital	Head of infectious disease department	1
	Head of respiratory department	1
	Head of radiology department	1
	TB clinic doctor	1
	Public health staff	1
	Laboratory technician	1
Township hospital	Outpatient doctor	1
	Outpatient Traditional Chinese Medicine doctor	1
	Township TB control officer	1
Village health station	Chief of station	1
	Doctor	1
Community health station	Chief of station	1
** Total**		17

Male: 11, female: 6.

### Analysis

A thematic approach was used for analyzing the data to address existing themes and identify emerging ones. Researchers involved in this study worked as a team to design the study, develop questions to guide the interviews and to analyze the data, which provided a form of triangulation. The MAXQDA 10 QDA tool was used for coding related themes and sub-themes which were continuously revised after analyzing each interview.

## Results

Because data analysis was a continuous process, several themes emerged and many factors were identified as challenges in different areas of TB control at the study site. Several interesting quotes are mentioned below. Respondents for each quote are identified based on their place and type of work. ‘Health managers’ are the staff involved in the non-clinical aspect of TB control while ‘health providers’ are the staff involved in controlling the clinical aspects of TB.

### Elderly population

Elderly patients were considered an obstacle in TB control by health providers in ZJG county. Participants reported that they had difficulties in convincing elderly patients to visit designated hospitals for TB diagnosis. Moreover, it was difficult for these elderly patients to comply with the DOTS treatment because they were less conscious of their health and did not understand the severity of the disease.

*“Elderly patients have different opinions about the medicines.’’(Health provider, town level)*

*“More and more people are getting old, so the source of infection is also widening.”(Health manager, CDC)*

Elderly patients were less compliant with the treatment, mostly due to the side effects. Doctors had a dilemma regarding the standard TB treatment for elderly patients because such patients often had other co-morbidities and were more susceptible to adverse drug reactions of antituberculosis treatments.

*“If we ask them (elderly patients) to take medicine, they always answer, ‘I don’t feel well after taking that medicine; then why are you pushing me to take that medicine again?’ So, as doctors, we really don’t know what to do.” (Health provider, designated hospital)*

*“For such patients (elderly), if they can’t eat food after taking a medicine, then I will say, ‘food is more important than taking medicine’. For such elderly patients, we can’t follow the standard antituberculosis treatment.”(Health provider, designated hospital)*

Elderly patients were also considered as a source of drug resistant TB. *“More focus should be put on the elderly patients and how drug resistance can be avoided among them. If they have drug resistant TB, there is no special place to separate them from their family members, as these patients prefer to stay at home. So it’s difficult to control further transmission.” (Health provider, designated hospital)*

Lack of knowledge about TB among elderly patients, co-morbidities, and susceptibility to the side effects of standard TB treatments were considered major issues faced by health providers working in hospitals and health management staff working in the field.

### Migrant workers

Health providers identified migrant workers as the major source of TB cases in the county and admitted that dealing with migrant workers was very difficult, as migrant workers were highly mobile, which made them hard to reach and difficult to monitor during treatment.

*“It (Incidence of TB) is because of the migrant workers. There are very few cases of TB among the local population.”(Health provider, village level)*

Following a referral from lower health facilities, migrant workers did not always report to the designated hospital. In such situations, health staff had to track them. However, temporary addresses and low levels of education made it difficult for health staff to find them.

*“Migrant workers couldn’t provide their exact address. They just give the name of the street. There are so many people living on that street. So how can we find them?”(Health manager, town level)*

Migrant workers always migrate to different cities and sometimes to different provinces in China. At the time of the Spring Festival, migrant workers usually return to their hometowns; this aggravated the problem of monitoring them during the treatment. The majority of health providers showed dissatisfaction regarding the monitoring of TB patients in ZJG county, especially migrant workers because they travel all over China. As a local health professional, it was hard for them to monitor such patients.

*“It’s not true that we don’t want to manage them (migrant workers). But actually the work of managing migrant workers doesn’t have clear boundaries. If we find a patient, we have to manage that patient till the end of treatment. How is it possible? If patients move from one place to another unknown place, we don’t have any way to monitor them.”(Health provider, designated hospital)*

Health providers showed willingness to treat migrant workers, but there was some confusion about their roles and responsibilities if these migrant workers leave their area of jurisdiction. Health providers and managers mentioned a wide range of challenges they faced in dealing with migrant workers.

*“In my opinion, these patients must be taken care of at the national level. But who will take care of them? It should be clear.”(Health manager, CDC)*

### Doctors’ motivation

Interviews with doctors revealed that although they considered referring and monitoring TB patients to be their responsibility and a contribution to public health, the incentives provided to them by the government for referring and monitoring TB patients were not enough. One respondent expressed the feeling that for doctors, such incentives were very minor and did not play any role in their motivation to refer and monitor TB patients. Instead, incentives were perceived as a tool to monitor doctors’ performance in managing TB patients.

*“I told you just now that this incentive is really nothing for us, very little, actually very little. But in this way, actually they have an assessment indicator in it.”(Health provider, designated hospital)*

On the issue of financial incentives for the doctors, one participant from the CDC agreed with the doctor’s opinion and felt that everyone in the health system cared about patients, but doctors involved in TB control were being neglected by policy makers.

*“There is not much motivation among doctors working in this risky field. You can imagine, if the doctor is not satisfied, how can the patient be satisfied?’’(Health manager, CDC)*

### Drug resistance and the need for new drugs

Doctors dealing with TB patients were unhappy with the current TB treatment options, because patients were getting more resistant to the first line drugs and there was not much they could do to improve the situation. Frustration over the lack of new drugs to treat TB was evident during the interviews with most of the respondents.

*“I want to say more about the medicine, because we doctors use medicine just like the weapons in a war. I know that our country is spending so much money on TB, but the discovery of new medicines is lagging far behind.”(Health provider, designated hospital)*

Drug resistance was clearly acknowledged as a ‘serious problem’ and the lack of new drugs and non-adherence to the treatment were cited by the doctors and the participants from the CDC, respectively, as being the major reasons for increased resistance.

*“In my area, the problem is with patients who do not comply with the treatment. After all, it is a 6 month treatment. Some of them take drugs for 2 months and find out that the condition has improved. Then they think that they are cured and don’t adhere to the treatment, resulting in higher risks for drug resistance.”(Health manager, town level)*

Health providers had a good understanding of reasons for drug resistance emerging among TB patients in the local context, but there was very little they could do to change the situation.

### Financial burden

According to the respondents, reimbursement provided by the government was an effective way to encourage patients to complete DOTS treatment. They felt that the reimbursement had a psychological effect on patients, convincing them that the government was paying attention to them. Most of the respondents thought that free diagnosis and treatment was enough to cover most of the TB patients’ expenses related to this disease.

*“Actually, patients do not need to spend a lot of money. Patients just need to spend a little money.”(Health provider, village level)*

When we asked the respondents about the causes of the financial burden on patients, medicine to protect their livers and hospitalization were noted as probable contributors to the burden. Prescribing liver protection medicine as a preventive measure was a common practice.

*“If they spend a lot of money, they must have been hospitalized. Under normal circumstances, they don’t need to spend so much money.”(Health provider, town level)*

Upon exploring the issue of unnecessary hospitalization practices by certain hospitals for financial benefit, doctors in the designated hospitals denied any unnecessary hospitalization practices in their hospitals.

*“Ren Mmin Yi Yuan (People’s County Hospital) does not have enough rooms to keep those patients (TB patients). So it’s impossible that the hospital will hospitalize them just to earn money.”(Health provider, designated hospital)*

Health providers were unaware of any heavy direct or indirect financial burden on uncomplicated pulmonary TB patients. Expenses for liver protection medication and CT scans were cited as the primary causes of the financial burden if there was one.

### Social stigma

Despite mass health education and health promotion, a social stigma regarding TB still existed in ZJG county. Some migrant workers were reported to go back to their hometowns after being diagnosed with TB. If factory owners knew about their health condition they might lose their jobs, therefore some migrant workers did not like to provide their real contact information which created more problems for health providers monitoring such patients.

*“After getting this disease (TB), some patients would lose their jobs. It is difficult for them to work again because they are regarded as the source of infection.”(Health provider, CDC clinic)*

The stigma existed not only among migrant workers. Some local urban residents were reluctant to accept the diagnosis of TB. One doctor told us that some patients were even worried that their TB status might create trouble for them if they had to take a flight.

*“If you call the patients, they usually feel annoyed. They think that what we say is nonsense. ‘Who told you that I have TB’? This attitude is sometimes problematic for us in performing the DOTS strategy.”(Health manager, designated hospital)*

### An integrated model of hospital–TB program collaboration in practice

Health providers at all levels considered a hospital-TB control program collaboration to be a win-win situation. The CDC had the edge in managing health programs while hospitals had the expertise to provide clinical care for patients.

*“They (designated hospitals) just manage the clinical parts. They say that only diagnosis is their job, and patient management is too complicated for them. It’s not their responsibility to manage the patients, so they just make the diagnosis. After that, they refer the patient to us (CDC). Now it has become a model.”(Health manager, CDC)*

The CDC had a limited capacity to deal with complex clinical cases of TB and hospitals were not capable of providing public health services. Therefore, this integrated model was considered a success by respondents both from the CDC and the designated hospitals.

*“In the field of clinics, they (hospitals) have more expertise, and on our side (CDC), we have more experience in the preventive aspects of the disease.”(Health manager, CDC)*

## Discussion

The objective of this qualitative study was to gain an in-depth understanding of health providers’ perceptions of current TB control programs and to identify challenges faced by them in rural China. Our study revealed that factors such as reimbursement, improvements in socioeconomic status of the population, and the standard of living resulted in improved health seeking behavior and adherence to treatment. Migrant workers and the elderly were repeatedly identified as the main obstacles in effective tuberculosis control in the county.

Elderly TB patients are a matter of concern for health providers both working at the CDC and in hospitals in ZJG county. Elderly patients have been repeatedly reported to have a lower treatment completion rate [9], [10]. According to our respondents, elderly patients are less health conscious than younger patients, so they have to put extra effort into convincing elderly patients to seek diagnosis and complete the treatment. Doctors in ZJG county were faced with a dilemma over the standard treatment for elderly patients because they usually have other co-morbidities and higher susceptibility to side effects of antituberculosis drugs; these results support the findings reported in other countries [11]–[13]. Previous studies have shown that pulmonary TB in the elderly is characterized by atypical symptoms, more extensive radiological lesions with lower zone preponderance, higher sputum positivity, more frequent co-morbidities, more frequent side effects and higher mortality [14]. Side effects of antituberculosis drugs on liver function are already known [15]. Elderly patients spend more money on liver protective medications, extra diagnostic tests and, in some cases, hospitalization. Another country with a large elderly population is Japan, where this problem is addressed using the ‘liaison critical pathway for tuberculosis’. Under this pathway, a network to support adherence to TB treatment is built for elderly patients, and health center staff have expanded the community DOTS in the elderly by establishing an effective community collaboration [13]. TB is complicated by other co-morbidities, so addressing underlying diseases and new complications is critical for successful management of TB among the elderly. Drug dosage needs to be adapted for elderly patients, keeping co-morbidities in mind. During the 1990s, a public health approach to TB control was developed with limited resources in mind. However, patient-centered care was a significant change from the DOTS strategy in 1990 to the Stop TB strategy in 2006 [16]. Findings from our study indicate that TB control strategies and treatment regimens need to be patient centered, especially in the case of elderly patients. China has the world’s largest aging population [17], and aging is an inevitable cause of a reduced immunological response. It is estimated that the proportion of the population over age sixty in China will grow from the current 10.9% to 35.8% in the next fifty years [18]; therefore, the number of TB cases and the degree of drug resistance in China might increase exponentially, putting a heavy economic burden on the country. NTP in China will have to prepare to adapt and develop new case-detection strategies, treatment guidelines and patient-centered care for the elderly population.

Developing countries such as China have a vast disparity in income among residents of different provinces and urban/rural areas giving rise to internal migration of people in search of better job opportunities and better income. Industrialized counties such as ZJG in the eastern part of China attract a large number of migrant workers. Migrant workers have been reported to have higher rates of TB compared to local residents in China [19]. Adherence to TB treatment is at the core of the 6–8 month-long DOTS regimen, but the ‘migratory’ nature of migrant workers has a negative effect on adherence. A study on adherence to DOTS treatment among migrant workers in the Shandong province of China found that 16% of smear-positive TB patients in the study were non-adherent to the treatment. Most of the migrant workers reside alone in cities, away from their spouses and families, lacking support from family members and suffering from an increased financial burden [20]. China’s restrictive ‘hukou’ (local residence permit) system has long been criticized for not giving equal opportunities to migrant workers to bring their families to urban areas, and such policies have a distinct effect on TB control too [21]. Migrant workers said that factors such as their poor economic status, lower level of education, and lower awareness about TB, along with health system-related factors such as complexities in the referral and diagnostic procedures, provided many challenges for them in accessing TB care. The financial burden of TB treatment is reported to be higher among poor migrant workers. This problem is partly being addressed in ZJG county by providing additional travel and nutrition subsidies to migrant workers, but such practices are not universal in China. Other measures, such as comprehensive health education prior to starting chemotherapy and special attention to adherence by primary health workers, especially during the intensive phase of treatment, are recommended as a better TB control strategy for migrant workers [20]. Identifying TB cases among the migrant population is crucial because factors such as their migratory nature, worse living conditions, poor financial status, and low awareness of TB lead to poor outcomes and result in increased numbers of TB cases, thereby helping the TB epidemic to thrive [22], [23]. Migrant workers coming from rural parts of China present a gloomy prospect for TB control in China [23].

China has a serious epidemic of MDR-TB associated with inadequate treatment in both the public and private health systems [24]. The prevalence of MDR-TB among new cases in China was 3.5 times the global median and nearly twice the global mean, but China has slower progress in detection and enrollment of MDR-TB cases [25]. At the ground level, drug resistance was also considered the most challenging problem faced by health providers. The reasons for developing resistance were cited as non-adherence to treatment; patients stopped taking drugs after 2-3 months of treatment following the disappearance of their symptoms. Factors such as previous treatment history, old age and poverty are strongly associated with the risk of drug resistance in the Chinese population [26], [27]. Prevention and control of drug-resistant TB should emphasize prompt case detection, and quality-assured drug susceptibility testing (DST) for those patients at high risk of resistance. Doctors involved in our study expressed their frustration with increased resistance to the first line drugs. One doctor said that he felt helpless in front of his patients due to the lack of new medicines. Doctors complained that developed countries were not giving enough weight to the discovery of new drugs for TB. Rapid emergence of MDR-TB stresses the importance of the development of new antituberculosis drugs; but despite the dire need, very little progress has been made, because these drugs are not profitable for pharmaceutical companies [28]. Developing countries such as China and India, who bear the biggest brunt of TB, should take the lead in the development of new drugs to equip health providers to fight drug resistance at the ground level.

A stigma associated with TB is reported from many parts of the world, including China [29]–[31]. A study from rural areas of the Inner Mongolia Autonomous Region of China highlighted the social stigma associated with TB, indicating that it influenced marriage prospects and social interactions within the community [32]. The stigma places TB patients at a social disadvantage, leading to further social isolation, reduced health-seeking behavior and poor adherence to therapy. Our respondents noted that the stigma was more common among migrant workers and elderly patients in ZJG county. Sometimes patients provided false information to hide their identity due to the stigma associated with TB. Migrant workers feared losing their jobs if they were found to have TB. TB has already been reported to cause heavy direct and indirect financial burden on patients [33], [34]. If TB patients lose their jobs because of the stigma, it will increase the financial burden on them. The stigma is also a contributing factor for patient-related delays in seeking timely TB care, because many patients remain in denial of their TB status; this increases the delay in starting treatment, resulting in increased spreading of the infection. The TB-associated stigma is one of the determinants of non-adherence to DOTS treatment, aggravating the epidemic of MDR-TB in China. Successful TB control programs are based on early diagnosis and effective treatment, which limit the transmission of the pathogen. Insufficient knowledge about TB might affect early diagnosis and care [22], so health providers and doctors should intensify efforts to educate the public about TB at the community level.

Under China’s NTP, patients are provided free diagnosis and treatment for TB. However, several studies from China have shown that TB patients have to bear a high financial burden, which influences their health seeking behaviors [35]–[39]. Recent studies on the financial burden of diagnosis and treatment on TB patients in rural China highlights that TB patients have to spend large amounts of money on indirect costs such as traveling, loss of working hours, etc. [39]. Considering the higher financial burden on migrant workers, additional travel and nutritional subsidies were provided to them in ZJG county. We would like to draw attention to the common practice of prescribing liver-protective medication regardless of the clinical profile of individual patients in ZJG county. Prescription of such medication is not recommended by the WHO, and it increases the financial burden on the patients, as such medications are not part of the free medication provided under the NTP.

Globally, the funding for TB control increased from US$3.4 billion in 2006 to US$4.8 billion in 2013. Despite this increase in funding, weak health systems are hampering the global endeavor to achieve the targets of TB control and other health MDGs [40]. In many parts of the world, hospitals are considered the weak link in global TB control efforts [41]. The PPM approach is an essential component of the WHO’s Stop TB Strategy. However, the success of PPM is usually compromised by the lack of specific guidelines and conflicts over the roles and responsibilities of collaborating partners. In our study, one CDC staff has been appointed to a designated hospital for better case management and to facilitate communication between the designated hospital and the CDC. The integrated model used in ZJG county can provide a good model for hospital and public health system collaboration throughout China. In particular, it can replace the ‘specialist model’ which has been shown to increase the financial burden on patients, aggravate delays and increase the risk of drug resistance [24], [42]. The integrated model is hailed as a model for the future of China’s TB control system because it provides the simplest health-care-seeking pathway by reducing delays and the financial burden on patients [42]. Unwanted hospitalization for financial incentives by hospitals has been reported in China [43]. However, according to the doctors of the designated hospitals in ZJG county, their hospitals were usually overcrowded so there was little chance of admitting patients without actual need. Respondents from both the CDC and designated hospitals acknowledged that the integrated model was a win-win situation for both of them, as it brought together the experience in clinical care and management from designated hospitals and the CDC, respectively.

For an effective referral system, motivated health providers and a robust TB information system are essential [40]. Use of an internet-based system and active follow-up by health providers substantially improves the case detection rate [43], [44]. Financial incentives to health providers improve their motivation to refer and monitor TB patients, but such incentives are inadequate in China [45]. DOTS treatment lasts for 6–8 months and suspected TB patients rely on health providers’ suggestions to seek health care, so acknowledging the role of health providers in TB control by increasing financial incentives might have a greater motivational impact on health providers at lower level health facilities.

Some of our respondents attributed better TB control in the county to the improved socioeconomic status of the local population. There is a strong association between changes in national TB incidence and changes in national socioeconomic indices and the general health status of the population [46]. Several local strategies used in ZJG county have proved advantageous, including the use of mobile X-ray vehicle scanning systems to do health check-ups among factory workers and school children to improve the case detection rate. One respondent from the CDC told us that they cannot use the leaflets provided by the Global fund because those leaflets are made according to the dispensary model which is used in major parts of China. This shows that, in countries such as China with diverse health systems, strategies based on national guidelines but tailored to the local context can be effective.

China’s NTP is deemed a successful TB control program [43]. However, new challenges in TB control are emerging in China. Our study sheds some light on these challenges. China’s attempt to control TB may be jeopardized unless these challenges are addressed.

## Conclusion

Through our study, we identified the elderly population and migrant workers as bottlenecks for TB control in ZJG county. Therefore, more resources should be devoted to increasing case detection and treatment completion rates among these populations. A global effort to develop new drugs, along with measures to control MDR-TB, needs urgent attention. We would also like to recommend an integrated PPM model of hospital and health system collaboration for TB control in China. TB control policies need to adapt to changing TB dynamics to develop better TB control programs in China and the rest of the world.
